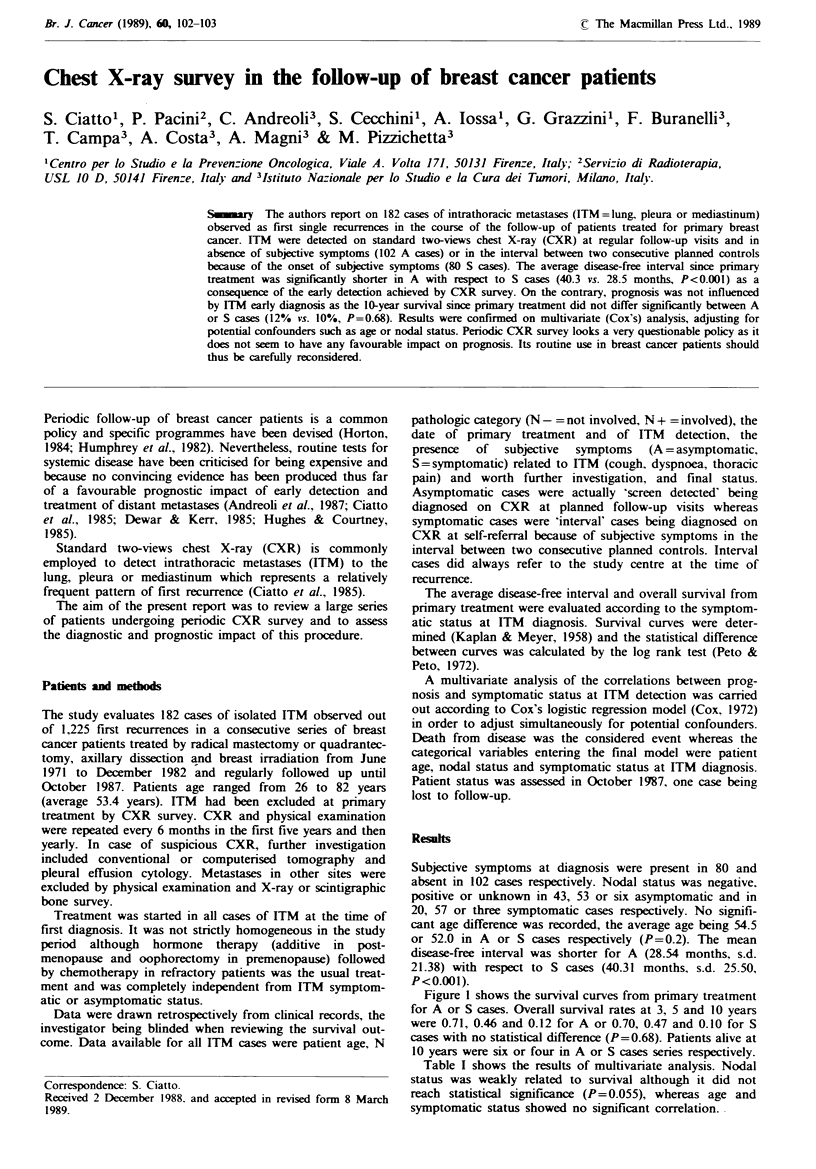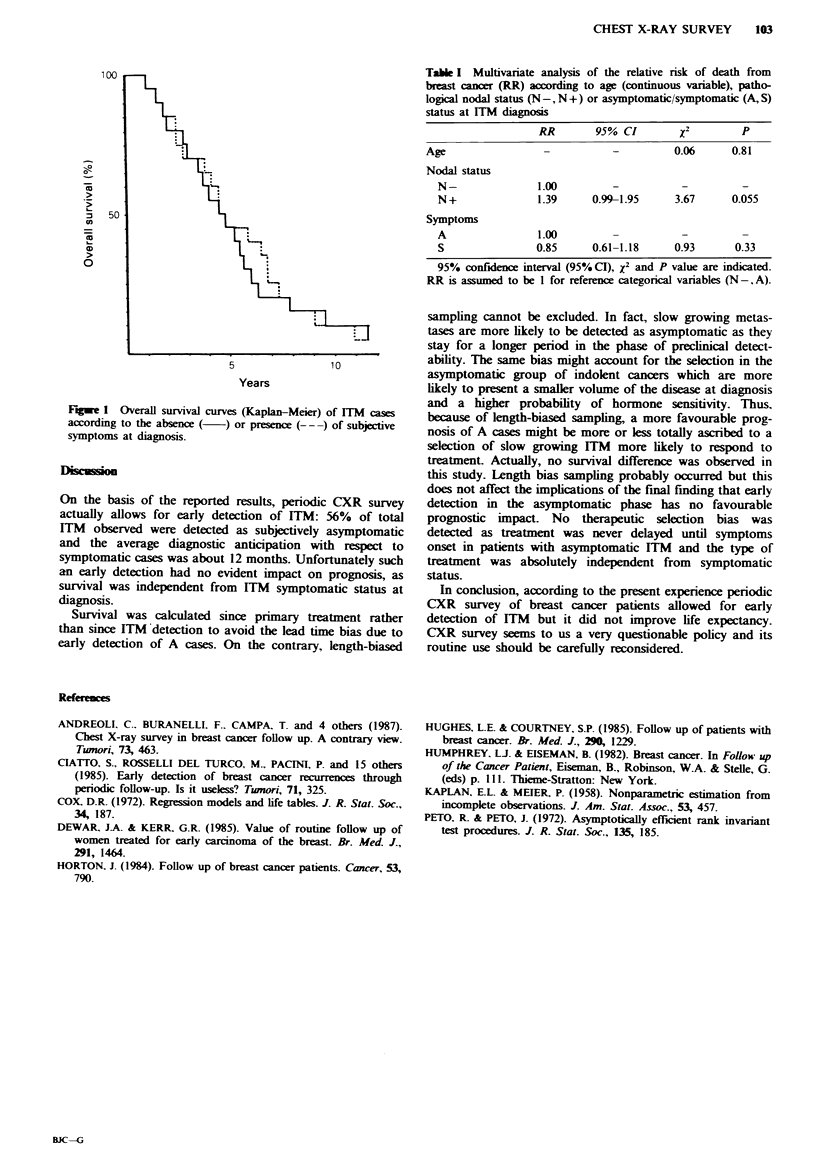# Chest X-ray survey in the follow-up of breast cancer patients.

**DOI:** 10.1038/bjc.1989.229

**Published:** 1989-07

**Authors:** S. Ciatto, P. Pacini, C. Andreoli, S. Cecchini, A. Iossa, G. Grazzini, F. Buranelli, T. Campa, A. Costa, A. Magni

**Affiliations:** Centro per lo Studio e la Prevenzione Oncologica, Firenze, Italy.

## Abstract

The authors report on 182 cases of intrathoracic metastases (ITM = lung, pleura or mediastinum) observed as first single recurrences in the course of the follow-up of patients treated for primary breast cancer. ITM were detected on standard two-views chest X-ray (CXR) at regular follow-up visits and in absence of subjective symptoms (102 A cases) or in the interval between two consecutive planned controls because of the onset of subjective symptoms (80 S cases). The average disease-free interval since primary treatment was significantly shorter in A with respect to S cases (40.3 vs. 28.5 months, P less than 0.001) as a consequence of the early detection achieved by CXR survey. On the contrary, prognosis was not influenced by ITM early diagnosis as the 10-year survival since primary treatment did not differ significantly between A or S cases (12% vs. 10%, P = 0.68). Results were confirmed on multivariate (Cox's) analysis, adjusting for potential confounders such as age or nodal status. Periodic CXR survey looks a very questionable policy as it does not seem to have any favourable impact on prognosis. Its routine use in breast cancer patients should thus be carefully reconsidered.


Br. J. Cancer (1989), 60, 102–103 © The Macmillan Press Ltd., 1989

### Chest X-ray survey in the follow-up of breast cancer patients

S. Ciatto[1], P. Pacini[2], C. Andreoli[3], S. Cecchini[1], A. Iossa[1], G. Grazzini[1], F. Buranelli[3], T. Campa[3], A. Costa[3], A. Magni[3] & M. Pizzichetta[3]

[1]*Centro per lo Studio e la Prevenzione Oncologica, Viale A. Volta 171, 50131 Firenze, Italy;* [2]*Servizio di Radioterapia, USL 10 D, 50141 Firenze, Italy and* [3]*Istituto Nazionale per lo Studio e la Cura dei Tumori, Milano, Italy.*

**Summary** The authors report on 182 cases of intrathoracic metastases (ITM = lung, pleura or mediastinum) observed as first single recurrences in the course of the follow-up of patients treated for primary breast cancer. ITM were detected on standard two-views chest X-ray (CXR) at regular follow-up visits and in absence of subjective symptoms (102 A cases) or in the interval between two consecutive planned controls because of the onset of subjective symptoms (80 S cases). The average disease-free interval since primary treatment was significantly shorter in A with respect to S cases (40.3 *vs.* 28.5 months, $P<0.001$) as a consequence of the early detection achieved by CXR survey. On the contrary, prognosis was not influenced by ITM early diagnosis as the 10-year survival since primary treatment did not differ significantly between A or S cases (12% *vs.* 10%, $P=0.68$). Results were confirmed on multivariate (Cox's) analysis, adjusting for potential confounders such as age or nodal status. Periodic CXR survey looks a very questionable policy as it does not seem to have any favourable impact on prognosis. Its routine use in breast cancer patients should thus be carefully reconsidered.

Periodic follow-up of breast cancer patients is a common policy and specific programmes have been devised (Horton, 1984; Humphrey *et al.*, 1982). Nevertheless, routine tests for systemic disease have been criticised for being expensive and because no convincing evidence has been produced thus far of a favourable prognostic impact of early detection and treatment of distant metastases (Andreoli *et al.*, 1987; Ciatto *et al.*, 1985; Dewar & Kerr, 1985; Hughes & Courtney, 1985).

Standard two-views chest X-ray (CXR) is commonly employed to detect intrathoracic metastases (ITM) to the lung, pleura or mediastinum which represents a relatively frequent pattern of first recurrence (Ciatto *et al.*, 1985).

The aim of the present report was to review a large series of patients undergoing periodic CXR survey and to assess the diagnostic and prognostic impact of this procedure.

### Patients and methods

The study evaluates 182 cases of isolated ITM observed out of 1,225 first recurrences in a consecutive series of breast cancer patients treated by radical mastectomy or quadrantectomy, axillary dissection and breast irradiation from June 1971 to December 1982 and regularly followed up until October 1987. Patients age ranged from 26 to 82 years (average 53.4 years). ITM had been excluded at primary treatment by CXR survey. CXR and physical examination were repeated every 6 months in the first five years and then yearly. In case of suspicious CXR, further investigation included conventional or computerised tomography and pleural effusion cytology. Metastases in other sites were excluded by physical examination and X-ray or scintigraphic bone survey.

Treatment was started in all cases of ITM at the time of first diagnosis. It was not strictly homogeneous in the study period although hormone therapy (additive in post-menopause and oophorectomy in premenopause) followed by chemotherapy in refractory patients was the usual treatment and was completely independent from ITM symptomatic or asymptomatic status.

Data were drawn retrospectively from clinical records, the investigator being blinded when reviewing the survival outcome. Data available for all ITM cases were patient age, N pathologic category (N− = not involved, N+ = involved), the date of primary treatment and of ITM detection, the presence of subjective symptoms (A = asymptomatic, S = symptomatic) related to ITM (cough, dyspnoea, thoracic pain) and worth further investigation, and final status. Asymptomatic cases were actually 'screen detected' being diagnosed on CXR at planned follow-up visits whereas symptomatic cases were 'interval' cases being diagnosed on CXR at self-referral because of subjective symptoms in the interval between two consecutive planned controls. Interval cases did always refer to the study centre at the time of recurrence.

The average disease-free interval and overall survival from primary treatment were evaluated according to the symptomatic status at ITM diagnosis. Survival curves were determined (Kaplan & Meyer, 1958) and the statistical difference between curves was calculated by the log rank test (Peto & Peto, 1972).

A multivariate analysis of the correlations between prognosis and symptomatic status at ITM detection was carried out according to Cox's logistic regression model (Cox, 1972) in order to adjust simultaneously for potential confounders. Death from disease was the considered event whereas the categorical variables entering the final model were patient age, nodal status and symptomatic status at ITM diagnosis. Patient status was assessed in October 1987, one case being lost to follow-up.

### Results

Subjective symptoms at diagnosis were present in 80 and absent in 102 cases respectively. Nodal status was negative, positive or unknown in 43, 53 or six asymptomatic and in 20, 57 or three symptomatic cases respectively. No significant age difference was recorded, the average age being 54.5 or 52.0 in A or S cases respectively ($P=0.2$). The mean disease-free interval was shorter for A (28.54 months, s.d. 21.38) with respect to S cases (40.31 months, s.d. 25.50, $P<0.001$).

Figure 1 shows the survival curves from primary treatment for A or S cases. Overall survival rates at 3, 5 and 10 years were 0.71, 0.46 and 0.12 for A or 0.70, 0.47 and 0.10 for S cases with no statistical difference ($P=0.68$). Patients alive at 10 years were six or four in A or S cases series respectively.

Table I shows the results of multivariate analysis. Nodal status was weakly related to survival although it did not reach statistical significance ($P=0.055$), whereas age and symptomatic status showed no significant correlation.

Correspondence: S. Ciatto.
Received 2 December 1988, and accepted in revised form 8 March 1989.

**Figure 1** Overall survival curves (Kaplan–Meier) of ITM cases according to the absence (——) or presence (- - -) of subjective symptoms at diagnosis.

**Table I** Multivariate analysis of the relative risk of death from breast cancer (RR) according to age (continuous variable), pathological nodal status (N −, N +) or asymptomatic/symptomatic (A, S) status at ITM diagnosis

| | *RR* | *95% CI* | $\chi^2$ | *P* |
|---|---|---|---|---|
| Age | – | – | 0.06 | 0.81 |
| Nodal status | | | | |
| N − | 1.00 | – | – | – |
| N + | 1.39 | 0.99–1.95 | 3.67 | 0.055 |
| Symptoms | | | | |
| A | 1.00 | – | – | – |
| S | 0.85 | 0.61–1.18 | 0.93 | 0.33 |

95% confidence interval (95% CI), $\chi^2$ and *P* value are indicated. RR is assumed to be 1 for reference categorical variables (N −, A).

### Discussion

On the basis of the reported results, periodic CXR survey actually allows for early detection of ITM: 56% of total ITM observed were detected as subjectively asymptomatic and the average diagnostic anticipation with respect to symptomatic cases was about 12 months. Unfortunately such an early detection had no evident impact on prognosis, as survival was independent from ITM symptomatic status at diagnosis.

Survival was calculated since primary treatment rather than since ITM detection to avoid the lead time bias due to early detection of A cases. On the contrary, length-biased sampling cannot be excluded. In fact, slow growing metastases are more likely to be detected as asymptomatic as they stay for a longer period in the phase of preclinical detectability. The same bias might account for the selection in the asymptomatic group of indolent cancers which are more likely to present a smaller volume of the disease at diagnosis and a higher probability of hormone sensitivity. Thus, because of length-biased sampling, a more favourable prognosis of A cases might be more or less totally ascribed to a selection of slow growing ITM more likely to respond to treatment. Actually, no survival difference was observed in this study. Length bias sampling probably occurred but this does not affect the implications of the final finding that early detection in the asymptomatic phase has no favourable prognostic impact. No therapeutic selection bias was detected as treatment was never delayed until symptoms onset in patients with asymptomatic ITM and the type of treatment was absolutely independent from symptomatic status.

In conclusion, according to the present experience periodic CXR survey of breast cancer patients allowed for early detection of ITM but it did not improve life expectancy. CXR survey seems to us a very questionable policy and its routine use should be carefully reconsidered.

### References

ANDREOLI, C., BURANELLI, F., CAMPA, T. and 4 others (1987). Chest X-ray survey in breast cancer follow up. A contrary view. *Tumori*, **73**, 463.

CIATTO, S., ROSSELLI DEL TURCO, M., PACINI, P. and 15 others (1985). Early detection of breast cancer recurrences through periodic follow-up. Is it useless? *Tumori*, **71**, 325.

COX, D.R. (1972). Regression models and life tables. *J. R. Stat. Soc.*, **34**, 187.

DEWAR, J.A. & KERR, G.R. (1985). Value of routine follow up of women treated for early carcinoma of the breast. *Br. Med. J.*, **291**, 1464.

HORTON, J. (1984). Follow up of breast cancer patients. *Cancer*, **53**, 790.

HUGHES, L.E. & COURTNEY, S.P. (1985). Follow up of patients with breast cancer. *Br. Med. J.*, **290**, 1229.

HUMPHREY, L.J. & EISEMAN, B. (1982). Breast cancer. In *Follow up of the Cancer Patient*, Eiseman, B., Robinson, W.A. & Stelle, G. (eds) p. 111. Thieme-Stratton: New York.

KAPLAN, E.L. & MEIER, P. (1958). Nonparametric estimation from incomplete observations. *J. Am. Stat. Assoc.*, **53**, 457.

PETO, R. & PETO, J. (1972). Asymptotically efficient rank invariant test procedures. *J. R. Stat. Soc.*, **135**, 185.